# Reduced Effective Connectivity in the Motor Cortex in Parkinson’s Disease

**DOI:** 10.3390/brainsci11091200

**Published:** 2021-09-12

**Authors:** Emanuela Formaggio, Maria Rubega, Jessica Rupil, Angelo Antonini, Stefano Masiero, Gianna Maria Toffolo, Alessandra Del Felice

**Affiliations:** 1 Department of Neuroscience, Section of Rehabilitation, University of Padova, Via Gustiniani 3, 35128 Padova, Italy; emanuela.formaggio@unipd.it (E.F.); stef.masiero@unipd.it (S.M.); alessandra.delfelice@unipd.it (A.D.F.); 2 Department of Information Engineering, University of Padova, Via Gradenigo 6/A, 35131 Padova, Italy; je.rupil@live.it (J.R.); giannamaria.toffolo@unipd.it (G.M.T.); 3 Parkinson and Movement Disorders Unit, Study Centre on Neurodegeneration (CESNE), Department of Neuroscience, University of Padova, Via Giustiniani 5, 35121 Padova, Italy; angelo.antonini@unipd.it; 4 Padova Neuroscience Center, University of Padova, Via Orus, 35128 Padova, Italy

**Keywords:** EEG, Granger causality, MultiVariate AutoRegressive models, global efficiency, sensory-motor integration

## Abstract

Fast rhythms excess is a hallmark of Parkinson’s Disease (PD). To implement innovative, non-pharmacological, neurostimulation interventions to restore cortical-cortical interactions, we need to understand the neurophysiological mechanisms underlying these phenomena. Here, we investigated effective connectivity on source-level resting-state electroencephalography (EEG) signals in 15 PD participants and 10 healthy controls. First, we fitted multivariate auto-regressive models to the EEG source waveforms. Second, we estimated causal connections using Granger Causality, which provide information on connections’ strength and directionality. Lastly, we sought significant differences connectivity patterns between the two populations characterizing the network graph features—i.e., global efficiency and node strength. Causal brain networks in PD show overall poorer and weaker connections compared to controls quantified as a reduction of global efficiency. Motor areas appear almost isolated, with a strongly impoverished information flow particularly from parietal and occipital cortices. This striking isolation of motor areas may reflect an impaired sensory-motor integration in PD. The identification of defective nodes/edges in PD network may be a biomarker of disease and a potential target for future interventional trials.

## 1. Introduction

Parkinson’s disease (PD) main clinical features are motor symptoms, which have a causal factor in dopamine depletion in the basal ganglia circuit [1]. This degenerative process translates in abnormally fast oscillatory activity expressed in the cortico-basal loop, with frequencies ranging up to 200–250 Hz [2]. At scalp level, this activity may be only recorded as beta frequency, due to the impedance and attenuation processes to which the travelling signal undergoes while passing through brain tissue, cerebrospinal fluid and bone. Pathological firing of the basal ganglia entrains oscillations within the supplementary motor area, lateral premotor cortex, as well as frontal areas in various electroencephalographic (EEG) frequency bands [3], with beta frequency band being the most affected.

More recently EEG modulation and connectivity profiling have been proposed to investigate PD progression stages [2]. Brain connectivity explores physical connections (structural connectivity), statistical dependencies (functional connectivity), and casual interactions (effective connectivity) between brain areas. In particular, effective connectivity studies the direct influence that one cortical area may exerts over another and describes the direction of the interaction.

A computational approach to estimate effective connectivity is Granger Causality (GC) [4] measured by MultiVariate AutoRegressive (MVAR) models. This interconnected representation of the brain can be conveniently treated as a network—i.e., in Mathematics a graph. Graph analysis constitutes a simple model to decode network structures, capturing their complexity and providing measures to characterize their topological features, functional organization, and/or the global or local properties of each node.

A network perspective may provide an intervention target on defective nodes: neurostimulation techniques, whether invasive or non-invasive, may restore cortico-cortical interactions [5,6,7]. Abnormalities in oscillatory activity reflect alterations in connectivity in the cortico-striato-thalamo-cortical circuit [8]. Impaired connectivity has also been described between cortical areas—e.g., prefrontal and motor cortex [9]. Most of these data stem from magnetic resonance imaging (MRI) studies, using either functional or structural connectivity methods [2]. Granger Causality applied to functional Magnetic Resonance Imaging (fMRI) has been deployed to study cortico-thalamo-striato-cortical connections in PD [10,11], confirming the weaker interaction between the caudate and the motor cortex.

Cortico-cortical interactions and their directionality in PD are less known [12]. MRI data focused almost exclusively on motor areas and demonstrated an increased causal connectivity from the left premotor cortex (PMC) to right primary motor cortex (M1). Applying GC to EEG data exploits a non-invasive, low-cost, and bed-side functional brain imaging technique [5,6,7].

The aim of the present study is to identify causal connectivity among cortical areas using source-waveforms in a cohort of PD individuals. Our hypothesis is that we may able to confirm MRI derived connectivity alterations in PD, with a particular involvement of the motor areas, with EEG-derived data.

## 2. Materials and Methods

### 2.1. Participants

Fifteen people with PD (9 M; age in the interval [60–83] years; mean disease duration 6.1 ± 4.6 years; mean L-dopa dose 450 ± 185.2 mg; subjects tested in ON-phase) were recruited from Movement Disorders Unit at the Neurology clinic of the Padova University Hospital (Table 1 and Table 2). All showed a bradykinetic phenotype. Inclusion criteria were: diagnosis of idiopathic PD within the last five years; stable dose of antiparkinsonian therapy for at least four weeks; off-medication motor Hoehn and Yahr 1–2 at enrolment. Exclusion criteria were: psychiatric disorder; benzodiazepine and neuroleptic treatment; Mini Mental State Examination (MMSE) < 23; contraindications to neurostimulation. Subjects were part of a randomized controlled trial to study the effect of neurostimulation and motor rehabilitation [13]. EEG data used for the present analysis were collected at baseline to avoid any interference with intervention. Control data from ten healthy volunteers (3 M; age in the interval [51–71] years) were obtained using the same EEG system. All participants provided written informed consent. The Ethics Committee of Padova University Hospital approved the protocol (protocol n. 3507/AO/15).

### 2.2. EEG Data

Five minutes of eyes-open resting state EEG signal (32-channels system; BrainAmp 32MRplus, BrainProducts GmbH, Munich, Germany) were acquired using an analogic anti-aliasing band pass-filter at 0.1–1000 Hz and converted from analog to digital using a sampling rate of 5 KHz. The reference was between Fz/Cz and ground anterior to Fz. The data were processed in Matlab R2018b (MathWorks, Natick, MA) using personalized scripts based on EEGLAB toolbox (http://www.sccn.ucsd.edu/eeglab (accessed on 1 September 2020) [14] and in Brainstorm (https://neuroimage.usc.edu/brainstorm (accessed on 1 September 2020) [15].

EEG data were band-pass filtered from 1 to 30 Hz using a even-order linear phase FIR filter avoiding phase-distortion and down-sampled using a sampling rate of 500 Hz by using Brainstorm software. Artefacts—i.e., eye movements, cardiac activity, and scalp muscle contraction—were visually identified and removed using independent component analysis implemented in EEGLAB (Infomax). The EEG data were further processed using a common average reference. Source-reconstructed time-series were obtained by using Brainstorm software [15]. First, a head model was created using a symmetric boundary element method based on the anatomy derived from the ICBM152 brain [16]. Time-series of neuronal activity were reconstructed using minimum norm algorithm. Sources were constrained to the cortex and the reconstructed time-series were projected onto regions of interest (ROIs) as defined by the Brodmann atlas [17], where time-series for voxels within a ROI were averaged and used for connectivity analysis. Eight ROIs were defined merging Brodmann areas (BA) in frontal, central, parietal and occipital regions, grouped separately for left and right hemisphere, defined as follow: central areas—BA 1, 2, 3, 4, 6; frontal areas—BA 8, 9, 10, 11, 44, 45, 46; parietal areas—BA 5, 7, 30, 39, 40, 43; occipital areas—BA 17, 18, 19. Time-series were divided into non-overlapping epochs of 2 s.

#### MVAR Model and Time-Domain Formulation of Granger Causality Index

Analysis was performed for each epoch using the MultiVariate Granger Causality Matlab^®^ Toolbox (MVGC) [18]. MVAR models represent a parametric method for discrete-time signal description. They extend to multiple time-series the AutoRegressive (AR) approach, which expresses the current value of a time-series as a weighted sum of its previous *p* values, plus a noise term. Under the assumption of having *m* stochastic processes $y_{i}\left(n\right)$; *i* = 1…, *m*; *n* = *p* + 1, …, *N* (in our study, m=8 as the number of ROIs, N=1000, i.e., the number of samples for each epoch), MVAR models allow to take into account and reconstruct the evolution of each of them in terms of its past history, the past history of all the other signals and a contribution from white noise. Our MVAR model, thus, consists of *m* linear equations, which can be conveniently written in matrix form as:

(1) $$y\left(n\right)=\sum_{k=1}^{p}A_{k}\cdot y\left(n-k\right)+u\left(n\right)$$

where y(n) is the vector of the *n*-th sample of the *m* processes, which has a deterministic component accompanied by the contribution of u(n) that represents the *m*-dimension vector of input white noise, with zero mean and diagonal covariance matrix $\Sigma_{u}$. The term *p* and the $A_{k}$ matrices are respectively the model order and the matrices of model coefficients. The model order defines the number of previous samples, from all the processes, that contribute to the expression of the current samples y(n); the weights of these samples are defined by the corresponding elements of the $A_{k}$ matrices, namely $A_{k}\left(i,j\right)$, and represents the influence of $y_{j}\left(n-k\right)$ on $y_{i}\left(n\right)$. The elements of the $A_{k}\left(i,j\right)$ matrices, together with the variances of the input noise appearing on the main diagonal of the $\Sigma_{u}$ matrix, represent the model parameters (their number is equal to 64×p+8) to be identified on EEG data of the eight ROIs.

According to Granger definition, a cause-effect relationship exists between two time-series $y_{i}\left(n\right)$ and $y_{j}\left(n\right)$, with *i* ≠ *j*, if the variance of the prediction error of $y_{i}\left(n\right)$, estimated with a MVAR model including all the *m* time-series of y(n) (unrestricted model), is lower than the one resulting from a MVAR model from which $y_{j}\left(n\right)$ is excluded (restricted model). Hence, the Granger causality from $y_{j}\left(n\right)$ to $y_{i}\left(n\right)$ can be measured with Geweke’s Granger Causality index, defined as:

(2) $$GC_{ij}=GC_{y_{j_{\to }y_{i}/y}}=\ln\frac{{\tilde{\sigma_{i}}}^{2}}{\sigma_{i}^{2}}$$

where ${\tilde{\sigma_{i}}}^{2}$ and $\sigma_{i}^{2}$ are the variance of predicted error $u_{i}\left(n\right)$ for restricted and unrestricted model regression, respectively.

For each subject of each group the analysis was firstly carried out epoch-wise. At first, the order of MVAR model was selected: Akaike parsimony criterion (AIC) was used for this purpose [19]. AIC optimum order was evaluated for each epoch with an upper bound of *p* = 20. The order that was most frequently selected (*p* = 9, i.e., 18 ms) was then fixed for all epoch/subjects. Equation (2) was evaluated for all the possible pairs of the eight ROIs defined in this study and the 8 × 8 matrix of GC indices represents causal strength among them—i.e., the (*i, j*) element of the GC matrix represents the strength of the influence that ROI j exerts on ROI i. F-test with Bonferroni correction was applied to check the statistical significance of each index (*p* < 0.000892): if the null hypothesis, that error variances were not significantly different, was accepted, the value of the corresponding GC coefficient was forced to zero for the current epoch.

The overall estimate of Granger connection magnitudes at a single subject level was obtained by averaging the GC matrices across all epochs. The percentage of epochs recognized as significant throughout the whole recording was also monitored for each subject. In order to graphically represent only the most relevant connections in the network, a threshold was computed using a surrogate data strategy: on the same dataset, the null distribution was determined using phase randomization—i.e., the dataset was transformed in frequency domain via fast Fourier Transform (FFT), randomly shuffled in order to change phase information and then back transformed in time domain via inverse FFT. This procedure was iterated 100 times, thus obtaining 100 realizations under the null hypothesis, and for each realization GC was computed. The threshold was fixed at the 95th percentile.

### 2.3. Graph Representation

Once the relevant connections between regions were estimated, the dynamics of global organization of the network was described using graph theory measures [20]. We mathematically represented the network as a graph—i.e., a set of vertices (or nodes) that represent our variables (ROIs) and a set of edges representing effective connections among them. The obtained graph was directed—i.e., the edges had a directionality—and weighted—i.e., the edges had variable weights measured by the magnitude of the Granger coefficients. The graph was exhaustively characterized by its adjacency matrix which shows the existing links among nodes. In our case of weighted directed graph, the adjacency matrix is non-symmetrical, thus allowing the differentiation between the incoming and outgoing connections of a node.

To obtain statistical inference regarding groups’ differences in non-threshold connectivity matrices (GC values), a Mann-Whitney-Wilcoxon test (*p* < 0.05) was performed. The significant connections, corresponding to *p* < 0.05, were plotted onto a MRI template using the BrainNet Viewer toolbox [21].

### 2.4. Graph Analysis

Graph analysis describes the network model properties by quantifying topologies of the network representation. Starting from the matrix, different topological measures can be derived for graph description [22]. To characterize the brain networks, parameters related to global efficiency and node strength were calculated by using an open-source toolbox (Brain Connectivity Toolbox, https://sites.google.com/site/bctnet/Home; accessed date: 1 December 2020). Global efficiency was computed as the inverse of the shortest weighted path length and evaluates the ability of the brain to rapidly combine specialized information from distributed brain regions. Structural networks usually are similarly organized and share a high global efficiency, whereas functional network have weaker connections between modules and consequently a weaker global efficiency [23,24]. Node strength was computed as the sum of weights of links connected to the node: the in-strength is the sum of inward link weights and the out-strength is the sum of outward link weights. In brain networks, this basic feature is not distributed homogeneously across the nodes since some nodes have a very high strength, marking them as putative network hubs [25].

A Mann-Whitney-Wilcoxon test (*p* < 0.05) was applied to statistically compare global efficiency and node strength between the two groups.

## 3. Results

**Figure 1:**
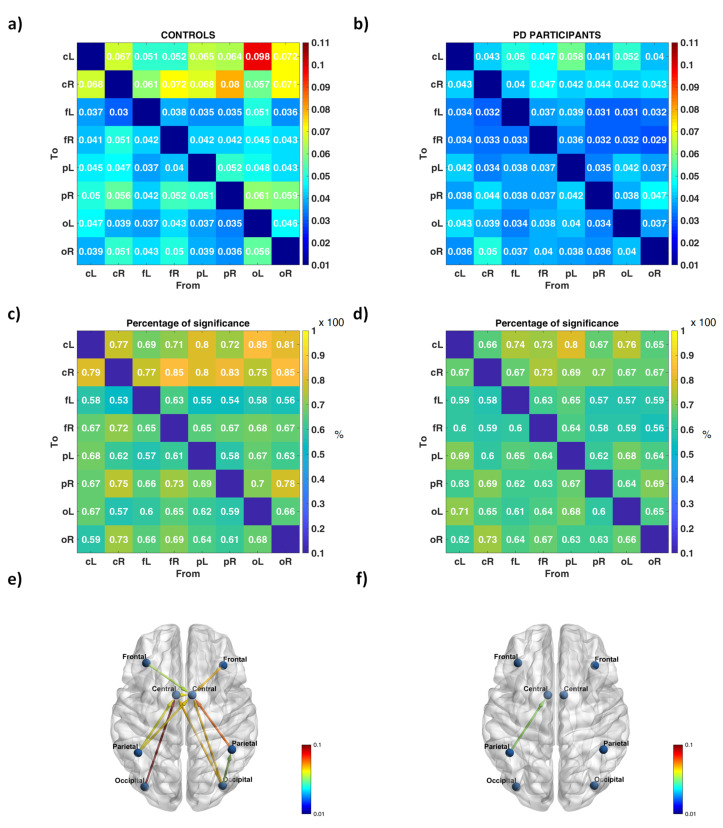
(**a**,**b**) Grand-average GC matrices for (**a**) controls and (**b**) PD participants. Element (i,j) - where i indicates the row and j the column - represents the weight of the connection from ROI j to ROI i. CR and cL denote central areas of right (R) and left (L) hemisphere, fR and fL frontal areas, pR and pL parietal areas and oR and oL occipital areas. The diagonal elements of the matrix are set to zero, since they represent connections from a ROI to itself (loops). (**c**,**d**) Grand-average percentage matrices for (**c**) controls (**d**) and PD participants: values in the matrix are reported as the result of the ratio, e.g., $0.77=\frac{77}{100}$ which corresponds to 77%. Element (i,j) represents the percentage of epochs where the connection from ROI j to ROI i was recognized as significant throughout the whole recording. (**e**,**f**) Connectivity networks for (**e**) controls and (**f**) PD participants. Only the most relevant connections are depicted, i.e., the ones recognized as significant in at least 79% of epochs. Circles stand for the eight ROIs and arrows for directed connections. Colorbars represent the magnitude of the brain connections.

Average results from GC analysis are summarized in Figure 1 for healthy controls (left panels) and PD participants (right panels): matrices of GC indices representing causal strength among the eight ROIs (upper panels), matrices of the percentage of epochs recognized as significant (medium panels), brain networks showing the connections significant in at least 79% of epochs (lower panels), where 79% is the threshold selected on surrogate data. Controls showed a higher number of connections; almost all of them, regardless of their origin, were directed to bilateral central regions (the motor cortex: BA 1, 2, 3, 4, 6) (Figure 1). The PD participants’ graph was overall poorer; only the connections from left parietal to left central node exceeded the threshold.

**Figure 2:**
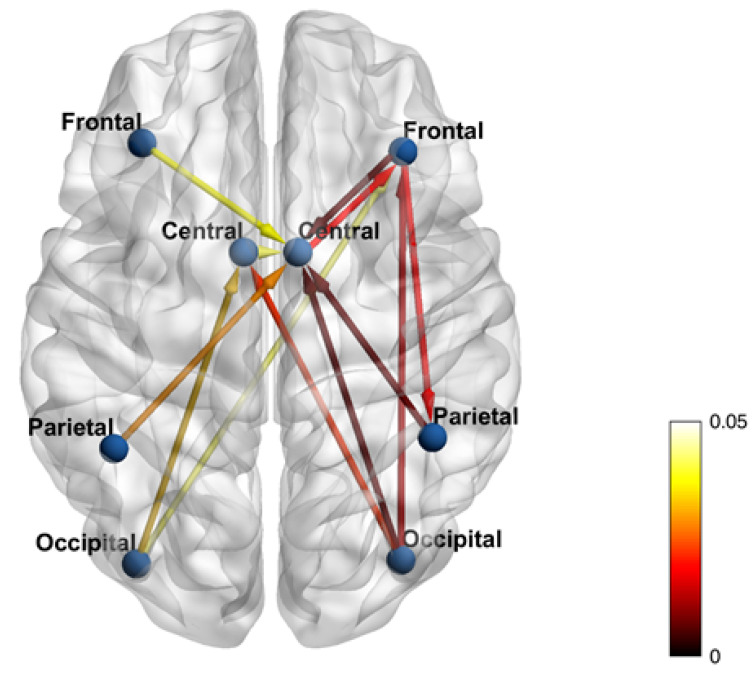
Statistically significantly different edges (*p* < 0.05) in controls vs. PD participants as calculated from the GC matrices. Edges values are derived from the Mann-Whitney-Wilcoxon test, which compares the non-threshold connectivity matrices between the two groups. Only the p-values of the significant connections (*p* < 0.05) were plotted onto the MRI template. Colorbar represents *p* values.

Comparison between the average GC matrices in the two groups (Figure 2) indicated significantly stronger connections (*p* < 0.05) in controls vs. PD participants for the edges from the left (L) frontal to the right (R) central node; from the R frontal to the R central and R parietal nodes; form L central to R central node; from the R occipital to R and L central, and to R frontal nodes; from L occipital to L central and R frontal nodes; from the L parietal to the R central node; and from the R parietal to the R central node.

Comparing Figure 1e with Figure 2 only 4 connections are not represented in Figure 1e (i.e., from R frontal node to R parietal node; from L occipital node to R frontal node, from R central node to R frontal node, from R occipital node to R frontal node). It has to be noted that the different threshold could affect the comparison: the 79% used to select significant connections within the group may be more stringent than the 0.05 *p*-value threshold used to select significant connections between the two groups.

**Figure 3:**
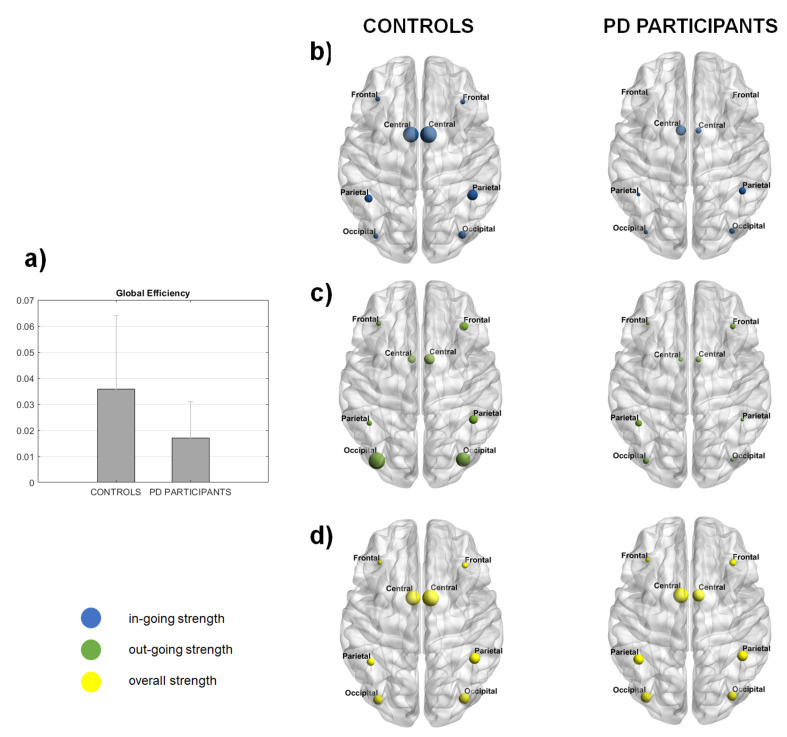
(**a**) Global efficiency and (**b**) in-going, (**c**) out-going (**d**) and overall strength for each node, in controls and PD participants. Indices were computed after applying the threshold at GC matrices for each subject. (**a**–**d**) represent the grand average. The error bar in (**a**) represents the standard deviation. The size of the node is proportional to the value of the index.

Global efficiency, a graph global measure expressed by a single value per network, was higher in controls (0.035) compared to PD participants (0.017) (Figure 3a), although the difference was not statistically significant. Node strength, a node-level measure based on the number of connections that a given node has with any other node of the network, showed higher values both for nodes with in-coming and out-coming information flow in controls (Figure 3b,c). The significant larger information flow to/from the motor nodes was clearly visible in both groups: for the in-coming information flow over R central node (*p* = 0.025), for the out-coming over R central (*p* = 0.0407) and R occipital (*p* = 0.0024) nodes, for the overall information flow over R frontal (*p* = 0.0327), central (*p* = 0.0043) and occipital (*p* = 0.0368) nodes (Figure 3b). The larger information flow to/from the motor nodes was clearly visible in both groups, especially for the in-coming flow (Figure 3b).

## 4. Discussion

Causal brain networks in PD, computed at a source level from EEG data, show overall poorer and weaker connections, calculated as a reduction of global efficiency, although the difference was not statistically significant, most likely due to the intra-subjects variability. Measuring cortical areas reciprocal interactions, we observed a striking isolation of motor areas in PD, especially for the information flow from the parietal and occipital areas to the motor cortices.

Our results highlight a critical parietal and occipital disconnection in PD and are in line with recent MRI data showing resting-state changes in dynamic functional connectivity and reduced number of transition between states in more severely compromised PD [26]. Other imaging studies suggest alterations of these areas: a Positron Emission Tomography (PET) investigation revealed that PD participants showed significantly lower relative cerebral blood flow in the bilateral occipital and posterior parietal cortex [27]. The parieto-occipital network oversees visual and sensory integration, processing the information to be incorporated into the motor program and action. The strict interplay between motor and visual system in the complex process of motor control appears impaired in PD [28,29] and is one of the core neuropsychological features of PD disease progression.

Impaired sensory-motor integration, reflected by the weakening of connections between parietal and central areas, is also confirmed by clinical signs: altered proprioception—i.e., the capability of recognizing body segment position in space—is considered a potential contributor to some peculiar PD clinical pictures—i.e., Pisa syndrome—and becomes even more evident as in the end stages of disease.

We observed a defective incoming information flow at the right motor area in PD. An interesting hypothesis rests on the differential function of dominant and non-dominant limb [30,31,32]. In reaching movements, the non-dominant arm appears better adapted for achieving accurate final positions and the dominant one for specifying initial trajectory features, such as movement direction and peak acceleration. According to this view, the visuo-spatial integration inflow to the right motor cortex, even at rest, may signal the more detailed visuo-spatial control exerted on the left arm, which reaches targets with more accuracy. In fact, people with PD show hypometria in reach-to-grasp tasks [33], interpreted as a deficit of sequential implementation of complex movements. Recent work reported also a difference in body representation during action in PD, which in fact rests, according to the authors, on complex cognitive integrative functions [34].

We noticed a reduction of network global efficiency in PD. The efficiency of a network is a measure of how efficiently it exchanges information across the whole set of edges. This finding suggest that overall information flow is defective in PD. Similar results have been observed in network topology analysis based on MRI data [35] and correlate with cognitive [36] and motor symptoms [37]. These observations confirm the reliability of GC computation based on EEG source analysis reconstruction; they may pave the way for a more widespread use of EEG data, which are part of clinical practice and low-cost, for this kind of analysis. Supporting this thesis, in our previous study [38], resting state relative power of the same PD group was compared to the relative power of a different group of healthy participants in each frequency band. All PD participants showed a statistically significant increase of beta rhythm over sensorimotor areas and a substantial decrease in delta range compared with healthy subjects (*p* < 0.05). These results are in line with emerging theories suggesting PD is characterized by excessive synchronization in the beta frequency band. The relationship between beta rhythm and clinical state is less clear but some studies have shown strong correlations with rigidity and bradykinesia at rest [39,40]. The average Montreal Cognitive Assessment (MoCA) score in PD participants was 24.79 [38], suggesting a slight degree of cognitive deficit. We did not analyze a single subject correlation between EEG power spectra and cognitive performance at MoCA. In fact, a progressive increase of slow (theta and delta) rhythms is a well known feature in PD. On this ground, the detection of beta band excess may be considered an even stronger finding considering the likely relative increase in this cohort of slow rhythms.

The abnormal oscillatory activity may be modulated by Deep Brain Stimulation (DBS) to stop the extrapyramidal tremor [41]. Based on the same concept, extradural motor cortex stimulation was evaluated in PD with the objective to entrain the pathological circuit at the cortical site [42]. Lastly, non-invasive brain stimulation techniques, namely repetitive transcranial magnetic stimulation (rTMS) and transcranial direct current/alternating stimulation (tDCS/tACS), have been deployed with the same rationale and to extend the efficacy of cognitive rehabilitation [13,43,44]. Given the results that non-invasive stimulation can reduce abnormal brain activity, results from the EEG connectivity could be potentially used to direct the stimulation and optimize restoration of connectivity.

Since recent work [45,46] provided direct evidence that scalp EEG can sense both cortical and subcortical signals, the promising results of this preliminary study support a future data acquisition with high-density EEG systems (≥64 channels), exploiting individual head models based on magnetic resonance imaging.

The choice of the quantification of causal interactions through the time domain formulation of Granger causality in the source-waveforms domain enabled us to overcome some interpretation caveats and common pitfalls that may arise when performing effective connectivity on scalp EEG [47]. Among them, (1) the large distance between EEG sensors and neural sources, and the spatial blurring effect of the skull on the electrical potential EEG distribution —i.e., volume conduction, which causes spurious correlation values between sensors; (2) the differences in signal-to-noise (SNR) ratio—i.e., impedances are equated as much as possible prior to data recordings, but in practice they cannot be equal for all sensors and stationary during the entire recording between signals, which can drive the asymmetries in the causality estimation; (3) common inputs (not included in the computational model), which can lead to spurious inference. Granger causality in the source domain allows to mitigate the adverse effect of volume conduction, also by discarding the interactions at $0^{\circ }$ phase and to putatively provide all the common inputs/generators of EEG scalp data. In our model, signals are a combination of the signal-of-interest and a superimposed noise to minimise the effect of variable SNR.

Eventually, to evaluate how much the system is fault tolerant and how much the communication is efficient, the causal interactions were modelled as the weighted edges of a graph: strength and global efficiency of the nodes—i.e., brain regions—of the graph were estimated and compared between PD participants and controls.

The use of threshold is a typical approach for eliminating the weakest connections. This methodology is still not universally accepted, because it may influence the number of links included in the graph analysis and could affect the indices extracted from the network. We used an automatic method based on surrogate and uncorrelated data that enables us to control for spurious findings.

Our study has some limitations. First, the accuracy of EEG source-waveforms reconstruction depends on a sufficient sampling of the surface potential field and increases as the sampling density and coverage increases [48]. Second, despite the method for source reconstruction is computationally cheap and easy to implement, it is based on ad-hoc assumptions and constraints: different assumptions embedded in the source localization method used to solve the inverse problem might lead to different results. Results might be also influenced by the choice of the brain parcellation and the method used to deal with the dipole orientation of all solution points in the same region of interest [49].

In summary, the use of MVAR models and a consolidated index such as the Granger Causality index in the time domain allowed to assess alteration in brain connectivity in PD participants. Future studies might finalize the analysis by using extended version of Granger causality, e.g., to account for the frequency features of the connections and minimize the volume conduction artefact—e.g., partial Granger causality [50], conditional Granger causality [51,52], directed transfer function [53] or partial directed coherence and their derivatives [54,55].

## 5. Conclusions

Our results provide evidence of an overall resting-state cortico-cortical reduced connectivity in PD: motor areas appear almost isolated, with a strongly impoverished information flow particularly from parietal and occipital cortices. The identification of defective nodes/edges of a network may provide a therapeutic target for future interventional trials.

## Figures and Tables

**Table 1:** Demographic characteristics of included participants.

Subject (S)	Age	Sex	Education
1	72 y	Male	16 y
2	64 y	Female	5 y
3	73 y	Male	13 y
4	79 y	Male	5 y
5	80 y	Female	13 y
6	69 y	Male	8 y
7	61 y	Female	11 y
8	75 y	Male	13 y
9	71 y	Male	17 y
10	63 y	Male	10 y
11	68 y	Male	17 y
12	60 y	Male	17 y
13	65 y	Female	5 y
14	66 y	Female	17 y
15	83 y	Female	5 y

**Table 2:** Clinical characteristics of included participants.

S	Duration	L-Dopa Dose	UPDRS III	Bradykinesia	Tremor	Axial Symptoms
1	2 y	800 mg	36	4	1	1
2	2 y	450 mg	28	6	0	1
3	1 y	200 mg	30	2	0	1
4	10 y	300 mg	31	4	2	0
5	3 y	300 mg	37	5	0	0
6	9 y	750 mg	35	1	0	2
7	11 y	600 mg	36	0	0	0
8	18 y	600 mg	29	3	0	1
9	6 y	400 mg	35	4	1	1
10	7 y	450 mg	32	2	0	2
11	2 y	300 mg	27	1	0	2
12	2 y	200 mg	38	2	1	1
13	8 y	400 mg	32	3	2	1
14	6 y	600 mg	35	3	0	0
15	4 y	400 mg	36	3	0	1

## Data Availability

The data presented in this study are available on request from the corresponding author. The data are not publicly available due to patients’ privacy.

## Abbreviations

The following abbreviations are used in this manuscript:

PD	Parkinson’s Disease
EEG	ElectroEncephaloGraphy
MVAR	MultiVariate AutoRegressive
MRI	Magnetic Resonance Imaging
fMRI	functional Magnetic Resonance Imaging
PMC	PreMotor Cortex
M1	Primary Motor Cortex
GC	Granger Causality
MMSE	Mini Mental State Examination
ROI	Region Of Interest
BA	Brodmann Areas
MVGC	MultiVariate Granger Causality
AIC	Akaike parsimony criterion
FFT	Fast Fourier Transform
tDCS/tACS	transcranial Direct Current/Alternating Stimulation
rTMS	repetitive Transcranial Magnetic Stimulation
